# Using Biosensors and Digital Biomarkers to Assess Response to Cardiac Rehabilitation: Observational Study

**DOI:** 10.2196/17326

**Published:** 2020-05-20

**Authors:** Hélène De Cannière, Christophe J P Smeets, Melanie Schoutteten, Carolina Varon, Chris Van Hoof, Sabine Van Huffel, Willemijn Groenendaal, Pieter Vandervoort

**Affiliations:** 1 Mobile Health Unit, Limburg Clinical Research Center (LCRC) Faculty of Medicine and Life Sciences Hasselt University (UHasselt) Diepenbeek Belgium; 2 Department of Future Health Ziekenhuis Oost-Limburg Genk Belgium; 3 Holst Centre imec the Netherlands Eindhoven Netherlands; 4 Center for Dynamical Systems, Signal Processing and Data Analytics (STADIUS) Department of Electrical Engineering (ESAT) KU (Katholieke Universiteit) Leuven Leuven Belgium; 5 Circuits and Systems (CAS) Department of Microelectronics Delft University of Technology (TU Delft) Delft Netherlands; 6 imec vzw Belgium Leuven Belgium; 7 Department of Cardiology Ziekenhuis Oost-Limburg Genk Belgium

**Keywords:** wearables, sensor, 6MWT, rehabilitation, cardiovascular

## Abstract

**Background:**

Cardiac rehabilitation (CR) is known for its beneficial effects on functional capacity and is a key component within current cardiovascular disease management strategies. In addition, a larger increase in functional capacity is accompanied by better clinical outcomes. However, not all patients respond in a similar way to CR. Therefore, a patient-tailored approach to CR could open up the possibility to achieve an optimal increase in functional capacity in every patient. Before treatment can be optimized, the differences in response of patients in terms of cardiac adaptation to exercise should first be understood. In addition, digital biomarkers to steer CR need to be identified.

**Objective:**

The aim of the study was to investigate the difference in cardiac response between patients characterized by a clear improvement in functional capacity and patients showing only a minor improvement following CR therapy.

**Methods:**

A total of 129 patients in CR performed a 6-minute walking test (6MWT) at baseline and during four consecutive short-term follow-up tests while being equipped with a wearable electrocardiogram (ECG) device. The 6MWTs were used to evaluate functional capacity. Patients were divided into high- and low-response groups, based on the improvement in functional capacity during the CR program. Commonly used heart rate parameters and cardiac digital biomarkers representative of the heart rate behavior during the 6MWT and their evolution over time were investigated.

**Results:**

All participating patients improved in functional capacity throughout the CR program (*P*<.001). The heart rate parameters, which are commonly used in practice, evolved differently for both groups throughout CR. The peak heart rate (HR_peak_) from patients in the high-response group increased significantly throughout CR, while no change was observed in the low-response group (F_4,92_=8.321, *P*<.001). Similar results were obtained for the recovery heart rate (HR_rec_) values, which increased significantly over time during every minute of recuperation, for the high-response group (HR_rec1_: *P*<.001, HR_rec2_: *P*<.001, HR_rec3_: *P*<.001, HR_rec4_: *P*<.001, and HR_rec5_: *P*=.02). The other digital biomarkers showed that the evolution of heart rate behavior during a standardized activity test differed throughout CR between both groups. These digital biomarkers, derived from the continuous measurements, contribute to more in-depth insight into the progression of patients’ cardiac responses.

**Conclusions:**

This study showed that when using wearable sensor technology, the differences in response of patients to CR can be characterized by means of commonly used heart rate parameters and digital biomarkers that are representative of cardiac response to exercise. These digital biomarkers, derived by innovative analysis techniques, allow for more in-depth insights into the cardiac response of cardiac patients during standardized activity. These results open up the possibility to optimized and more patient-tailored treatment strategies and to potentially improve CR outcome.

## Introduction

Cardiovascular diseases are the most prevalent noncommunicable diseases worldwide. The American College of Cardiology Foundation, the American Heart Association, and the European Society of Cardiology consider cardiac rehabilitation (CR) to be a key component within current disease management strategies, making millions of cardiac patients eligible for rehabilitation [1-3]. Unfortunately, CR programs worldwide are characterized by low implementation rates, as 33%-71% of eligible patients are not referred [4]. Notwithstanding, participation in a CR program has shown to increase cardiorespiratory fitness, thereby improving physiological responses to physical effort [5]. The improvement in functional capacity is clinically relevant as it not only improves quality of life in patients, but also serves as a powerful predictor for mortality [6]. These benefits of CR on mortality, morbidity, and quality of life have been studied comprehensively in several meta-analyses [7-10]. Moreover, the beneficial effects of CR seem to be even more pronounced when functional capacity is increased to a larger extent [11-14]. Although previous research showed that exercise-based CR has beneficial and clinically relevant effects on functional capacity, a large variability on the response to training is seen among CR patients. Recent studies have shown that *responders* are characterized by lower baseline peak VO_2_ (peak oxygen uptake) values and a reduced baseline ejection fraction, while *nonresponders* have an impaired chronotropic competence, which predicts poor training response [15-17]. Other potential contributing factors to poor training response include adherence rates, exercise dose, functioning of the autonomic system, or comorbidities [18]. Nevertheless, little is known about the mechanisms causing the large variability. Therefore, Gevaert et al stated that future research needs to focus on studying these contributing factors in order to generate the best response to CR [18]. Moreover, research focusing on changes occurring during CR, and not only before or after completing the program, can contribute in the development of a more patient-tailored CR program.

The aim of this study was to investigate the difference in cardiac response, a measure of chronotropic response, between patients that showed a clear improvement in functional capacity and patients that only showed a minor improvement following CR therapy. This was done by using data captured with a wearable electrocardiogram (ECG) device during a standardized activity. Moreover, innovative analysis techniques were used to derive digital cardiac biomarkers allowing an in-depth analysis of heart rate behavior during a standardized activity test.

## Methods

### Study Design

A total of 129 cardiovascular patients, who were enrolled in a multidisciplinary CR program in a single tertiary-care center (Ziekenhuis Oost-Limburg [ZOL], Genk, Belgium) and representative of the typical CR population, were included. Patients over the age of 18 years with heart failure and reduced ejection fraction, with heart failure and preserved ejection fraction, and with a left ventricular ejection fraction less than or equal to 55% were eligible for the study. Patients with an inability to exercise due to orthopedic or neurological limitations were excluded from the study. The goal was to investigate the different levels of response to exercise intervention during a standardized CR program. The 6-minute walking test (6MWT) was used to follow up on the improvement in functional capacity in the course of the CR program. A wearable device was used to collect ECG data during the 6MWT. A descriptive analysis of the longitudinally collected wearable data was performed to identify patterns or trends in the dataset. To distinguish the response to rehabilitation, patients were assessed as being within a low-response and a high-response group based on a median split for the increase in distance walked throughout the CR program. Therefore, two groups with an equal number of patients were created based on the level of improvement in functional capacity measured by the 6-minute walking distance. Patients who increased more than 90 meters after completing the CR were referred to as the high-response group, while the low-response group consisted of patients who increased less than 90 meters. The study complied with the Declaration of Helsinki, and the local ethical committee approved the study protocol. All subjects gave written informed consent prior to study participation.

### Multidisciplinary Cardiac Rehabilitation Program

Patients were referred to the multidisciplinary CR program following a cardiovascular-related hospital admission. The 15-week program consisted of 45 ambulatory rehabilitation sessions at a frequency of three 1-hour sessions per week. Both resistive and aerobic exercises were included in the program. Additionally, dietary sessions, psychological support, and social consultations were included in the multidisciplinary program. By standard, a cardiopulmonary exercise test (CPET) was performed at baseline and at end-of-study to assess functional capacity. A total of 14 low-response group patients out of 45 (31%) and 21 high-response group patients out of 45 (47%) had a CPET at both baseline and end-of-study. The heart rate achieved at 90% of ventilator threshold during the CPET was chosen as the target heart rate during aerobic training. If no CPET data were available, target heart rate was set at 50%-80% of the maximal heart rate. Aerobic training consisted of 30-40 minutes, in total, of aerobic exercise on bicycle, hand bike, treadmill, and/or stepper. Resistive training was performed at 50%-80% of one repetition maximum and consisted of three sets of 15 repetitions on both the leg and arm press. Training intensity was increased every 2 weeks based on patient improvement according to the standard clinical practice of CR in our study center.

### Experimental Protocol and Sensor Technology

Demographics, clinical data, medical therapy, and echocardiography data were collected from the electronic medical record. A 6MWT was performed at baseline (ie, start of rehabilitation program). Four follow-up 6MWTs were performed every 3 weeks, resulting in five 6MWTs in total. The compliance rate with the rehabilitation program between every 6MWT for both groups was calculated. Patients were expected to follow three rehabilitation sessions per week, which is equal to nine rehabilitation sessions between two consecutive 6MWT measurements. If patients attended nine rehabilitation sessions between consecutive 6MWTs, a compliance rate of 100% was obtained. The 6MWT was performed according to a standardized protocol [19]. The distance walked after 6 minutes was recorded and was used to check functional capacity during CR. During the 6MWT, all enrolled patients were equipped with a wearable device. The wearable device was equipped with the MUlti SEnsor IC (MUSEIC) chip, supporting a wide range of sensor modalities, including ECG (512 Hz sample frequency) and accelerometer data (32 Hz sample frequency) (imec the Netherlands, Eindhoven, the Netherlands) [20]. The electrodes were positioned according to lead II of Einthoven’s triangle [21]. Prior to the 6MWT, patients were at rest for 5 minutes to record their resting heart rate. Additionally, a recuperation phase of 5 minutes was included after the 6MWT to record recovery heart rate.

### Preprocessing and Calculation of Static and Dynamic Heart Rate Parameters

The signal was divided into three parts: a 5-minute resting phase, a 6-minute walking phase, and a 5-minute recuperation phase. First, the artefacts present in the ECG signals were automatically detected and removed by means of the algorithm proposed by Varon et al [22]. Next, an algorithm performed the initial automatic R-peak detection [23] and incorrect detections were visually corrected. The R-peaks were then used to generate the heart rate by dividing the signal into 2-minute or 16-second windows with a 4-second stride, from which the heart rate parameters were derived. The heart rate parameters derived from the 2-minute windows were used for further analysis. A distinction was made between two types of these digital cardiac biomarkers. The static, commonly used heart rate parameters that comprise the information of specific periods of time during a standardized activity are placed into a single output parameter, whereas the other digital cardiac biomarkers represent the evolution of heart rate throughout the entire time span of a standardized activity test.

The resting heart rate (HR_rest_) was calculated by taking the mean heart rate during the final 20 seconds of the resting period. The peak heart rate (HR_peak_) was calculated by taking the mean heart rate obtained during the final 10 seconds of the walking phase. For the recovery heart rate (HR_rec_), the mean heart rate during every minute of recuperation following the 6MWT (ie, HR_rec1_, HR_rec2_, HR_rec3_, HR_rec4_, and HR_rec5_) was calculated. Moreover, to study whether HR_peak_ was influenced by the difference in effort among patients, HR_peak_ was corrected for the distance walked by dividing HR_peak_ by distance (HR_peak-dist_), as described previously [24]. These heart rate parameters, commonly used in practice, were calculated for each measurement session separately. The accelerometer data has been used to estimate the effort during the walking phase of the 6MWT. Effort has been previously used as a measure of physical activity intensity [25-27]. The effort has been calculated for the full 6-minute-test duration using the following formula:


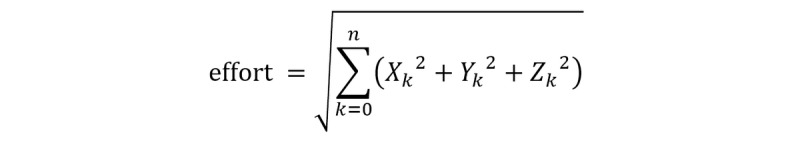


in which n is the total number of accelerometer sample points considered and X_k_ is a vector representing the acceleration along the x-axis, while the other axes are represented by Y_k_ and Z_k_ vectors, respectively.

Four different models were used to study the heart rate behavior during a standardized activity for both patient subgroups: one-term, two-term, two-term with added coefficient, and quadratic polynomial models. The goodness of fit was determined by calculating the coefficient of determination (R-squared). The model with the best fit was used to study the heart rate behavior. The coefficients of the best fits—the digital cardiac biomarkers—were studied for differences between both groups.

### Study Endpoints

This study focused on investigating the difference in cardiac response, reflected by changes in commonly used heart rate parameters and digital cardiac biomarkers, between patients with a clear increased functional capacity and patients with only a minor improvement after completing CR.

### Statistics

Continuous variables are expressed as mean (SD), if normally distributed, or as median (IQR), if nonnormally distributed, and dichotomous data are expressed as n (%). Normality was checked by the Shapiro-Wilk statistic. Categorical data were expressed as numbers and percentages and compared with the Fisher exact test. Continuous variables were compared between groups with the Student *t* test or the Mann-Whitney U test as appropriate. A two-way mixed analysis of variance (ANOVA) investigated the effect of magnitude of improvement on the progression of heart rate measures throughout a CR program. Results are expressed as df_main_, df_error_, F, and partial η². df_main_ indicates degrees of freedom for the simple main effect and df_error_ indicates degrees of freedom for the error term. F indicates that we are comparing results to an F distribution and partial η² is a measure of effect size. If significant, a repeated-measures ANOVA was performed to analyze the simple main effect over time and univariate analysis was performed to analyze the main effect of response groups. An independent *t* test was performed to compare cardiac biomarkers between groups at specific moments in time. Outliers were assessed by inspection of a boxplot for values greater than 3 box-lengths from the edge of the box and were removed from analysis. The statistical significance was always set at a two-tailed probability level of <.05. Statistics were performed using SPSS version 24.0 (IBM Corp).

## Results

### Demographics and Baseline Population

Of the 129 patients that consented to participate, 89 (69.0%) completed the total study protocol. Out of 129 patients, 40 (31.0%) were excluded from analysis upon failure to complete the CR program due to health-related problems, lack of motivation, and work or family commitment (see Multimedia Appendix 1). The 89 patients who completed the study protocol were subdivided into two groups based on their improvement in functional capacity throughout the CR. The high-response group consisted of 45 patients who improved more than 90 meters throughout the CR, while the low-response group consisted of 44 patients who improved less than 90 meters. Baseline characteristics of both groups are provided in Table 1. There was no statistical difference between the groups with respect to the demographics and baseline characteristics, except for diabetes. More often, patients in the low-response group suffered from diabetes compared to the high-response group (24.4% vs 2.3%, *P*<.01). The two-way mixed ANOVA showed a similar compliance rate between both subgroups throughout the rehabilitation program (F_3,255_=1.03, *P*=.99). The low- and high-response groups showed a similar average compliance rate of 88.0% and 85.8%, respectively, during the initial 3 weeks of the program, which did not significantly change throughout the remaining 12 weeks of the program.

### Functional Capacity

Patients showed an increase in functional capacity based on both the results of the CPET measurements and the results of the 6MWTs. Only 14 patients out of 44 in the low-response group (32%) and 21 patients out of 45 in the high-response group (47%) performed both a CPET at baseline and at end-of-study. Although not statistically significant, an increase of 2.23 mL kg^-1^ min^-1^ in peak VO_2_ was seen between baseline and end-of-study for the low-response group (14/44, 32%, *P*=.10). The same group showed a mean significant increase of 55 meters in 6MWT distance between baseline and end-of-study (*P*<.001). The high-response group showed a significant increase of 5.10 mL kg^-1^ min^-1^ in peak VO_2_ (21/45, 47%, *P*<.001) and 147 meters in 6MWT distance between baseline and end-of-study (*P*<.001).

**Table 1:** Baseline characteristics.

Variable		Low-response group^a^ (n=45)	High-response group^b^ (n=44)	*P* value
**Demographics**				
	Gender (male), n (%)	30 (67)	35 (80)	.23
	Age (years), mean (SD)	64 (9)	63 (10)	.83
	Height (m), mean (SD)	1.72 (0.09)	1.73 (0.09)	.44
	Body surface area (m²), mean (SD)	1.96 (0.19)	1.93 (0.19)	.45
	Active smoker, n (%)	12 (27)	6 (14)	.19
	Left ventricle ejection fraction (%), mean (SD)	47 (12)	44 (14)	.18
	Cardiac resynchronization therapy, n (%)	2 (4)	2 (5)	>.99
**Reason for referral, n (%)**				
	Myocardial infarction	14 (31)	9 (20)	.33
	Heart failure	11 (24)	10 (23)	>.99
	Coronary artery bypass grafting	6 (13)	8 (18)	.57
	Percutaneous coronary intervention	4 (9)	2 (5)	.68
**Comorbidities, n (%)**				
	Atrial fibrillation	10 (22)	12 (27)	.63
	Hypertension	16 (36)	22 (50)	.20
	Dyslipidemia	20 (44)	19 (43)	>.99
	Diabetes	11 (24)	1 (2)	.004
**New York Heart Association class, n (%)**				
	Class I	11 (24)	14 (32)	.62
	Class II	24 (53)	20 (45)	
	Class III	9 (20)	10 (23)	
**Medications, n (%)**				
	Angiotensin converting enzyme inhibitor	25 (56)	25 (57)	>.99
	Beta-blocker	33 (73)	32 (73)	>.99
	Diuretics	19 (42)	20 (45)	.83
Baseline CPET^c^ peak VO_2_^d^ (mL/kg∙min), mean (SD)		17.2 (5.3)	16.8 (4.9)	.72
Baseline 6MWT^e^ distance (m), mean (SD)		496 (95)	473 (97)	.25
**Compliance rate (%), mean (SD)**				.99
	Baseline to first measurement	88.0 (17.0)	85.5 (13.9)	
	First to second measurement	87.7 (18.5)	85.7 (14.1)	
	Second to third measurement	84.5 (17.9)	81.9 (20.6)	
	Third to end-of-study measurement	85.4 (21.8)	83.5 (24.9)	

^a^This group consisted of patients who improved less than 90 meters throughout the cardiac rehabilitation.

^b^This group consisted of patients who improved more than 90 meters throughout the cardiac rehabilitation.

^c^CPET: cardiopulmonary exercise test.

^d^VO_2_: peak oxygen uptake.

^e^6MWT: 6-minute walking test.

### Commonly Used Static Heart Rate Parameters

**Figure 1:**
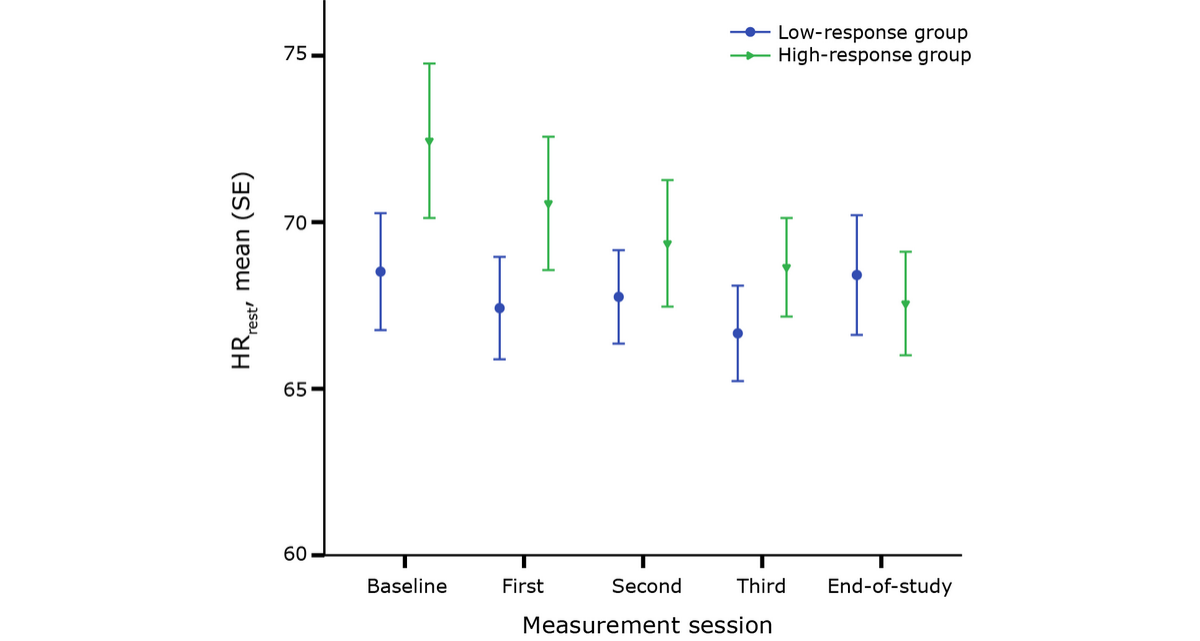
Resting heart rate (HR_rest_) for each group throughout cardiac rehabilitation.

To study the difference in cardiac response between the two subgroups, commonly used heart rate parameters were derived from the ECG data. Hereto, HR_rest_, HR_peak_, and HR_rec_ were analyzed. For these parameters, the differences between the two subgroups for every session and the differences in progression throughout CR were studied. Figure 1 shows the evolution of HR_rest_ throughout CR for both groups. A decreasing trend of HR_rest_ in the high-response group is observed. In addition, ANOVA did not show a difference in the evolution of HR_rest_ throughout the CR program. Nor was a difference seen in HR_rest_ between the high- and low-response groups at any point in time.

**Figure 2:**
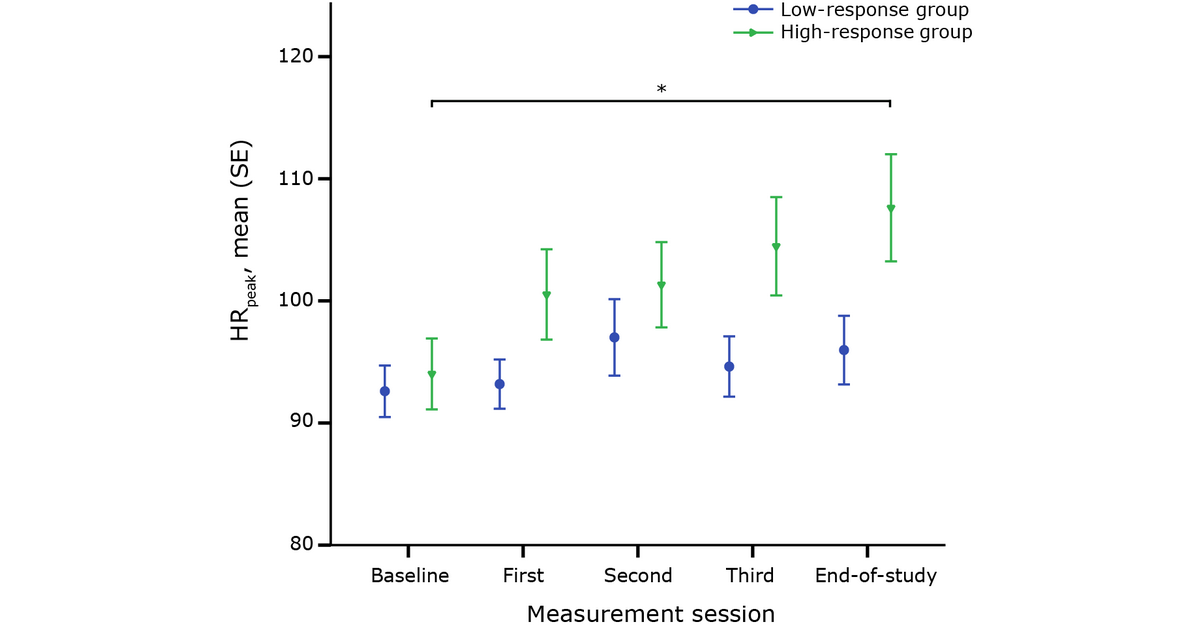
Maximum heart rate (HR_peak_) for each group throughout cardiac rehabilitation. *denotes a significant change over time.

Secondly, the evolution in HR_peak_ across five sessions and, more specifically, the difference between both subgroups was investigated. Figure 2 shows the change in HR_peak_ for both the high- and low-response groups throughout CR.

**Figure 3:**
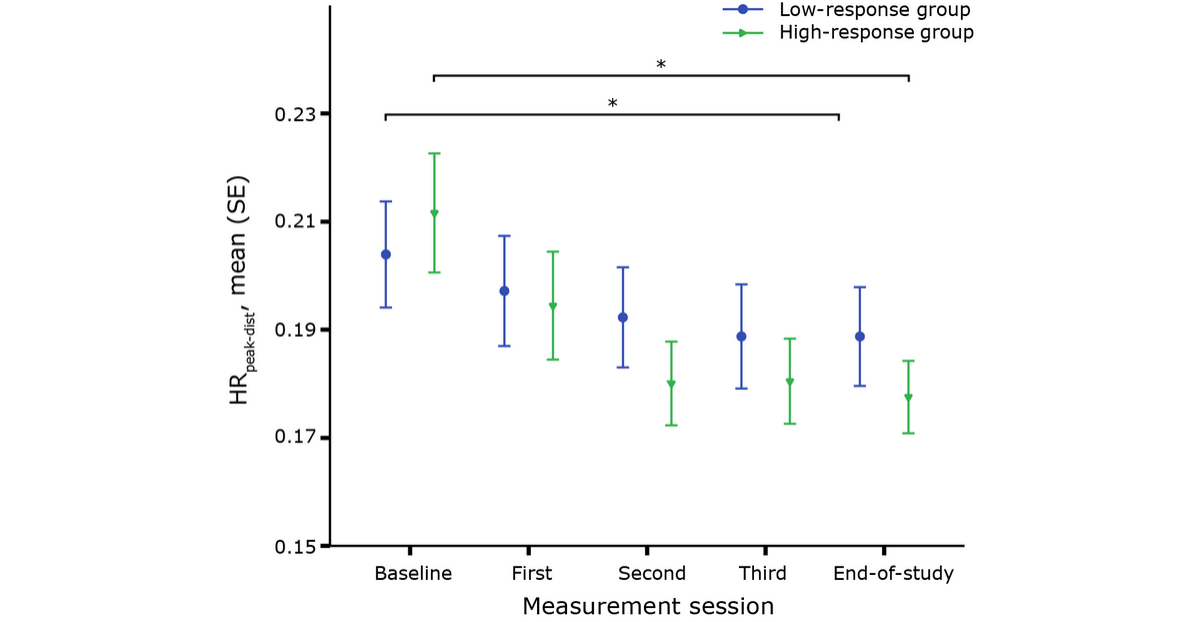
Peak heart rate corrected for distance (HR_peak-dist_) for each group throughout cardiac rehabilitation. *denotes a significant change over time.

The two-way mixed ANOVA showed that the evolution of HR_peak_ throughout CR differed between the high- and low-response groups (F_4,216_=3.3, *P*=.01, partial η²=.058). HR_peak_ of the high-response group increased significantly throughout rehabilitation, while HR_peak_ of the low-response group remained approximately the same (F_4,92_=8.321, *P*<.001). Although the evolution differed between both groups, no difference in HR_peak_ was seen at any point in time. HR_peak_ was corrected for distance to study whether the effort shown by patients during the 6MWTs had an influence on HR_peak_. In Figure 3, both subgroups show a significant decrease in HR_peak-dist_. However, the decrease in HR_peak-dist_ was larger for the high-response group compared to the low-response group. Additionally, a summary measure of the accelerometer data was used to compare the effort between the low- and high-response groups. Both subgroups showed a similar increase in effort throughout rehabilitation and no difference in effort between the groups was seen at any point in time (F_4,344_=.668, *P*=.61).

**Figure 4:**
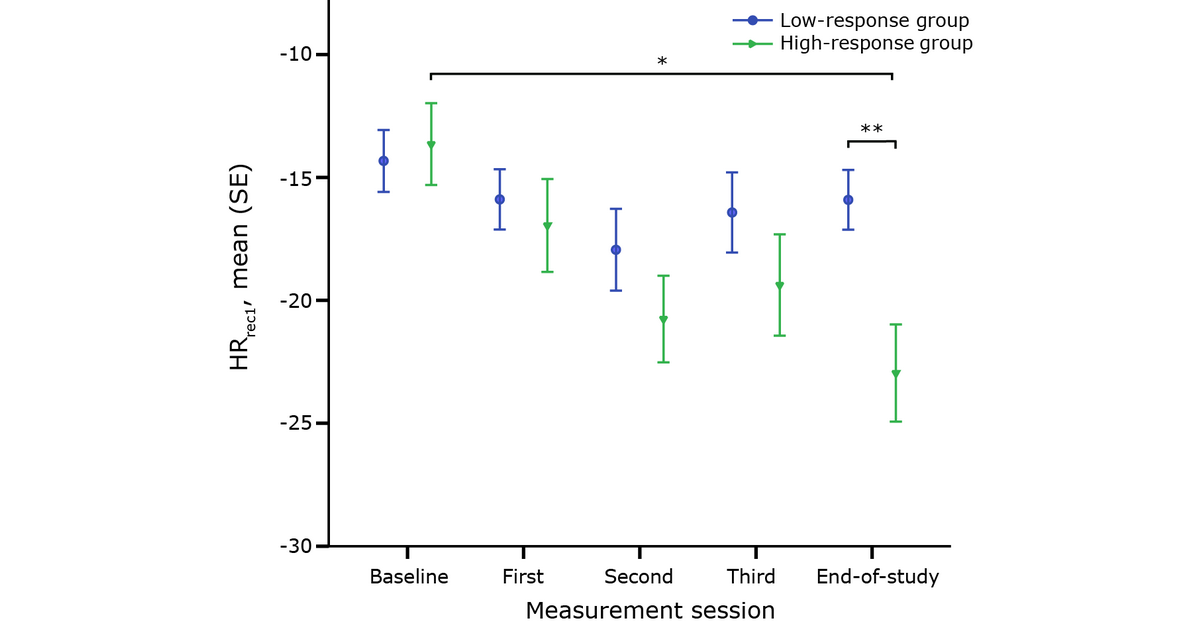
Heart rate recovery during the first minute (HR_rec1_) after the 6-minute walking test for each group throughout cardiac rehabilitation. *denotes a significant change over time; **denotes a significant difference in HR_rec1_ during a measurement session between both groups.

The change in HR_rec1_ after the 6MWT throughout CR is shown in Figure 4. The evolution of HR_rec_ throughout CR differed among the response groups according to ANOVA for every minute of recuperation (see Table 2).

**Table 2:** Results from the two-way, mixed-model, analysis of variance (ANOVA) for the heart rate recovery (HRrec) during the first 5 minutes after the 6-minute walking test (6MWT).

HR_rec_^a^	df_main_^b^	df_error_^c^	F^d^	Partial η²^e^	*P* value^f^	*P* value^g^
HR_rec1_	4	212	5.172	.089	.001	<.001
HR_rec2_	4	212	7.288	.121	<.001	<.001
HR_rec3_	4	212	6.634	.111	<.001	<.001
HR_rec4_	4	212	6.092	.103	<.001	<.001
HR_rec5_	4	208	3.967	.071	.03	.002

^a^HR_rec_: heart rate recovery; each number in this column represents every minute of recuperation following the 6MWT.

^b^df_main_: degrees of freedom for the simple main effect.

^c^df_error_: degrees of freedom for the error term.

^d^Indicates that we are comparing to an F distribution.

^e^Partial η²: a measure of effect size.

^f^Significance level for the hypothesis of no time effect × group effect.

^g^Significance level for the hypothesis of no time effect.

The HR_rec_ during every minute of recuperation increased throughout CR for the high-response group, while no change was observed in the low-response group (HR_rec1_: *P*<.001, HR_rec2_: *P*<.001, HR_rec3_: *P*<.001, HR_rec4_: *P*<.001, and HR_rec5_: *P*=.02). This evolution in HR_rec_ throughout CR resulted in a higher HR_rec_ in the high-response group at the end of the study (HR_rec2_: *P*=.02, HR_rec3_: *P*=.02, HR_rec4_: *P*=.03, and HR_rec5_: *P*=.02). In other words, patients from the high-response group will recuperate faster at the end of CR.

### Digital Cardiac Biomarkers to Capture the Dynamic Behavior of Heart Rate

The dynamic behavior of heart rate during a standardized exercise and subsequent recovery phase were investigated to further understand the difference in cardiac response between the groups. Hereto, four different models were fitted to the heart rate data. The quadratic polynomial fit obtained the best goodness of fit and was extracted from the heart rate data; the resulting coefficients, a_poly_ and b_poly_, were analyzed. Both coefficients determine the shape and steepness of the curve, thereby characterizing the speed of heart rate increase during the 6MWT and, thus, the response of the heart to exercise. These innovative digital cardiac biomarkers were studied for differences between the groups during every session, as well as for the difference in their progression throughout CR.

**Figure 5:**
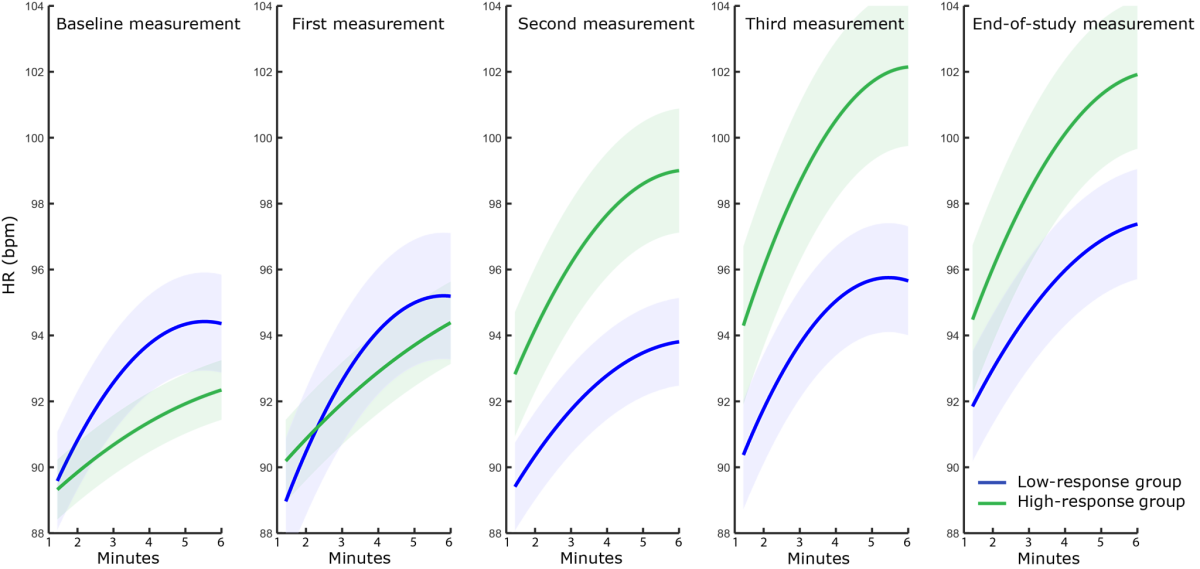
Mean quadratic polynomial fit to the changes in heart rate (HR) during all sessions while walking. The line represents the mean fit and the shadows represent the SD. bpm: beats per minute.

Figure 5 shows the mean quadratic polynomial fit (f(x) = a_poly_x² + b_poly_x + c) to the heart rate behavior during all five 6MWTs performed throughout the CR program. The behavior of heart rate measured during the walking phase evolved differently throughout CR in both groups. This is reflected in the difference in evolution for the polynomial coefficients, a_poly_ and b_poly_, between the groups (F_4,96_=5.691, *P*=.008, partial η²=.133 and F_4,160_= 4.302, *P*=.01, partial η²=.175). The higher values of a_poly_ and b_poly_ during baseline and the subsequent 6MWT in the low-response group indicate that the heart rate of these patients increases faster at the start of CR (-.00013 vs -.000027 and .065 vs .022, *P*<.001). However, toward the end of CR, the heart rate of the high-response group shows an increase and eventually catches up with the low-response group, showing a similar heart rate behavior during walking.

**Figure 6:**
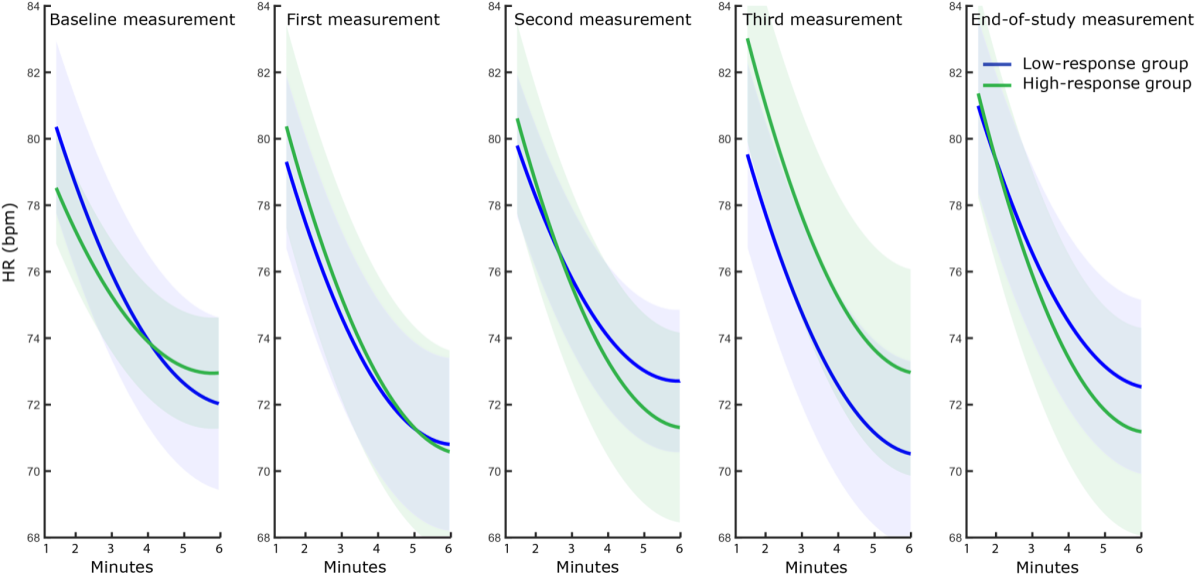
Mean quadratic polynomial fit to the changes in heart rate (HR) during all sessions while recuperating. The line represents the mean fit and the shadows represent the SD. bpm: beats per minute.

The behavior of heart rate during the subsequent recovery phase was analyzed for both groups (see Figure 6). The coefficient, b_poly_, extracted from the polynomial fit, showed a different evolution throughout CR between the groups (F_4,236_=3.367, *P*=.01, partial η²=.054). More specifically, the behavior of heart rate during recovery changed over time for the high-response group, while the low-response group showed no change in heart rate behavior during recovery (*P*<.001).

## Discussion

### Principal Findings

The findings of this observational study indicate that cardiac response to exercise in patients following a CR program plays a role in the level of their response to training in terms of distance walked. To our knowledge, this is the first study to describe the longitudinal follow-up of a CR patient population using wearable sensor technology during a repeated, standardized, submaximal activity test. Our 3-month follow-up period allows for novel in-depth insights into the cardiac response at rest, during exercise, and during recovery. Cardiac response is one of the possible confounders affecting the response to CR in patients. Therefore, investigating this cardiac response in a typical CR program aids in understanding the mechanisms behind different response rates. The wearable sensor technology enabled continuous monitoring of heart rate to derive both traditional heart rate parameters, commonly used in practice, and innovatively derived parameters that can function as digital cardiac biomarkers. The descriptive analysis of this longitudinally collected dataset investigated the difference in cardiac response between two patient populations following CR, who are respectively characterized by low and high improvement in functional capacity during CR.

### Evolution of Commonly Used Static Heart Rate Parameters Throughout Cardiac Rehabilitation

Previous research showed that the response to training depends on the cardiac output or chronotropic response, as this determines the increase in muscle blood flow during exercise [17,28]. Cardiac patients often depend on increasing their heart rate during exercise, as an increase in stroke volume is often limited due to the impaired cardiac output [29]. Heart rate parameters measured before, during, and after exercise contain information on how the heart responds to exercise. First, the effect of CR on the evolution of HR_rest_, HR_peak_, and HR_rec_ in patients showing a high or low response to exercise intervention was analyzed. These heart rate parameters, which are commonly used in practice, comprise the heart rate–related information from the resting, walking, and recuperation phases, respectively, into one single value.

HR_peak_ is a cardiac biomarker that evolves differently throughout CR for both response groups. The high-response group showed an increase in HR_peak_, while the low-response group showed no significant change. At a first glance, these results might appear to be opposite of the results from previous research [30-32], which showed that an increase in functional capacity is accompanied by a decrease in HR_peak_. However, they studied the heart rate response during a maximal exercise test, while in this study patients performed a submaximal test. The increase in HR_peak_ that we observed is accompanied by an increased distance. Hence, it is difficult to conclude whether the increased HR_peak_ is a result of the CR program or the fact that the walking distance also increases. Previous research showed that exercise intolerance causes a limitation in cardiac response in cardiac patients, as they achieved a higher heart rate compared to controls during submaximal exercise at a similar workload [33,34]. Effort is a measure of the walking intensity during the 6MWT. Both groups showed a similar effort. Although there was an increase in effort during the 6MWT throughout the CR, only the *response* group was characterized by an increase in HR_peak_. This indicated that the cardiac response in the *nonresponse* group was limited, leading to exercise intolerance throughout the CR program. Additionally, a correction was made for the distance. After correcting HR_peak_ for distance, a significant decrease in HR_peak-dist_ was seen for both groups. Thus, the results indicate that a cautious interpretation of heart rate parameters is necessary when studying the effects of CR on cardiac response during a submaximal exercise test.

The difference between the two groups in heart rate recovery, as captured by HR_rec_, was investigated. According to Qiu et al, HR_rec_ measured after the 6MWT is considered to be a powerful prognostic indicator in cardiovascular disease [35]. Cardiovascular patients are characterized by a slower HR_rec_ due to an attenuated autonomous nervous system. This decrease in HR_rec_ can be partially restored, as following a CR program improves the impaired recovery [36]. The results showed that HR_rec_ changed differently throughout the CR program for each group. In addition, our study showed no differences between the absolute HR_rec_ values during the first four sessions, but indicated a difference in HR_rec_ between the groups during the last session. The faster recuperation toward the end of CR could indicate the positive effect on the attenuated vagal reactivation within the high-response group, while this effect is absent in the low-response group.

To summarize, these heart rate parameters show that the effect of CR on the different response groups is also reflected in differences in cardiac response. The cardiac system of the high-response group adapts better to exercise throughout CR compared to the low-response group.

### Evolution of Digital Cardiac Biomarkers Throughout Cardiac Rehabilitation

To further understand the difference in cardiac response, the dynamic behavior of heart rate during the 6MWT and subsequent recuperation phase was also investigated. This innovative type of heart rate analysis allows in-depth insights by reflecting changes within shorter time spans during a standardized activity test. The heart rate response is representative of the ability of the autonomic nervous system to meet the hemodynamic demands during exercise. The heart rate acceleration at the onset of exercise is often modelled by an exponential curve [37]. Previous research showed that biphasic and sigmoidal curves are suitable to model heart rate behavior due to the increase in sympathetic activity following vagal withdrawal [38,39]. The choice to model the heart rate with exponential and polynomial curves was based on both literature and the heart rate behavior during the 6MWT, which was characterized by a steep increase followed by a steady-state phase as seen in this CR population.

The heart rate behavior during the 6MWT and subsequent recuperation phase evolved differently between the groups throughout CR. The increase in heart rate during the walking phase was steeper in the low-response group at baseline, indicating that in this phase of the CR the autonomous nervous system of these patients is characterized by a superior response to exercise in comparison to the high-response group. In the course of the CR program, the increase in heart rate steepened for the high-response group, eventually catching up with the low-response group. These results are similar to the findings of Schmid et al and Jorde et al, who indicated that the heart rate slope was blunted in subjects with an impaired cardiac response [17,40]. The ability of the *response* group to increase the heart rate slope, and the fact that the heart rate slope remained unchanged in the *nonresponse* group, indicated that an impaired cardiac response could lead to an impaired response to exercise training. Schmid et al showed that nonresponders to exercise training showed poor heart rate recovery, indicative of a disturbed cardiac autonomic status [17]. Our results confirm these findings, as both groups also showed a different evolution in heart rate behavior during the recuperation phase. The high-response group patients recuperated faster toward the end of CR, while the patients in the low-response group showed no change in recuperation rate.

To summarize, this study shows novel differences between groups, as the evolution in heart rate changes differently throughout the CR. Continuous measurements using wearable sensor technology enables the collection of traditional, commonly used heart rate parameters, but also of digital cardiac biomarkers representative of heart rate behavior during and after activity. The latter parameters were derived using a polynomial curve fitting technique, which is an innovative approach to capture heart rate evolution. Our research showed that these digital cardiac biomarkers can differentiate between the low- and high-response groups at baseline; hence, they, together with the traditional heart rate–related parameters, are valuable tools to use in short-term follow-up. Moreover, the results contribute to the development toward a more patient-tailored treatment strategy. Future research should focus on the role of these heart rate–related parameters in predicting outcome. The ability to improve and complement short-term follow-up by using these innovative techniques could make it possible to adjust treatment strategy in time and optimize outcome.

### Limitations

This study is an observational study analyzing the characteristics of a typical CR population; as patients were not randomized into different groups, the results should be interpreted as hypothesis generating. A low number of patients received both a baseline and end-of-study CPET measurement. These missing values do not influence the outcome of the study, as a submaximal exercise test is used to determine the progression in functional capacity throughout CR. There are some limitations to performing a median split to divide a patient population into two groups. However, data in this observational study was investigated in the search for trends upon which to base future randomized research. Another point of discussion is that the 6MWT is an effort-dependent test and a greater increase in HR_peak_ could be a consequence of higher effort. Therefore, effort derived from the data collected by the triaxial accelerometer was used to determine whether the high-response group was characterized by a higher effort in comparison to the low-response group. Hills et al state that acceleration is proportional to the net external force involved in an activity and, therefore, more directly reflects the energy cost associated with movement [41]. Moreover, effort is a parameter that is often used in VO_2_-max estimations during submaximal exercise [42,43]. Therefore, if the high-response group would have reached a higher percentage of their maximal exercise capacity during the 6MWT, this would have been reflected in a higher effort obtained during the 6MWT; however, this was not the case.

### Conclusions

Following CR is, without any doubt, beneficial for cardiovascular patients. However, some patients benefit more from CR as they show a larger improvement in functional capacity in comparison to other patients. This study shows the following:

1. Continuous measurements using wearable sensor technology during standardized activity give novel insights into cardiac response between different response groups.
2. Patients showing a larger increase in functional capacity are characterized by a better improvement in cardiac response. This is in contrast to patients showing a low response to exercise intervention.
3. Innovative analysis approaches allowed us to study the difference in heart rate behavior between the response groups in more detail, showing differences in cardiac response at baseline.
4. The results from this study can be used in future research to investigate whether the outcome of CR can be predicted in order to adjust treatment strategy. Moreover, it is a first step toward the development of a more patient-tailored CR program.

## Appendix

Multimedia Appendix 1 Flowchart containing the intervention measures, dropout reasons, and number of patients. 6MWT: 6-minute walking test.

## Abbreviations

6MWT: 6-minute walking test
ANOVA: analysis of variance
CPET: cardiopulmonary exercise test
CR: cardiac rehabilitation
df_error_: degrees of freedom for the error term
df_main_: degrees of freedom for the simple main effect
ECG: electrocardiogram
FWO: Research Foundation, Flanders
HR_peak_: peak heart rate
HR_peak-dist_: peak heart rate corrected for the distance walked
HR_rec_: recovery heart rate
HR_rest_: resting heart rate
LCRP: Limburg Clinical Research Program
LSM: Limburg Sterk Merk
MUSEIC: MUlti SEnsor IC
UHasselt: Hasselt University
VLAIO: Agentschap Innoveren en Ondernemen
VO_2_: peak oxygen uptake
ZOL: Ziekenhuis Oost-Limburg
