# Supplementary figures and images for: Using Biosensors and Digital Biomarkers to Assess Response to Cardiac Rehabilitation: Observational Study
Source: J Med Internet Res. 2020 May 20;22(5):e17326. doi: 10.2196/17326 (PMC7270861; doi:10.2196/17326)

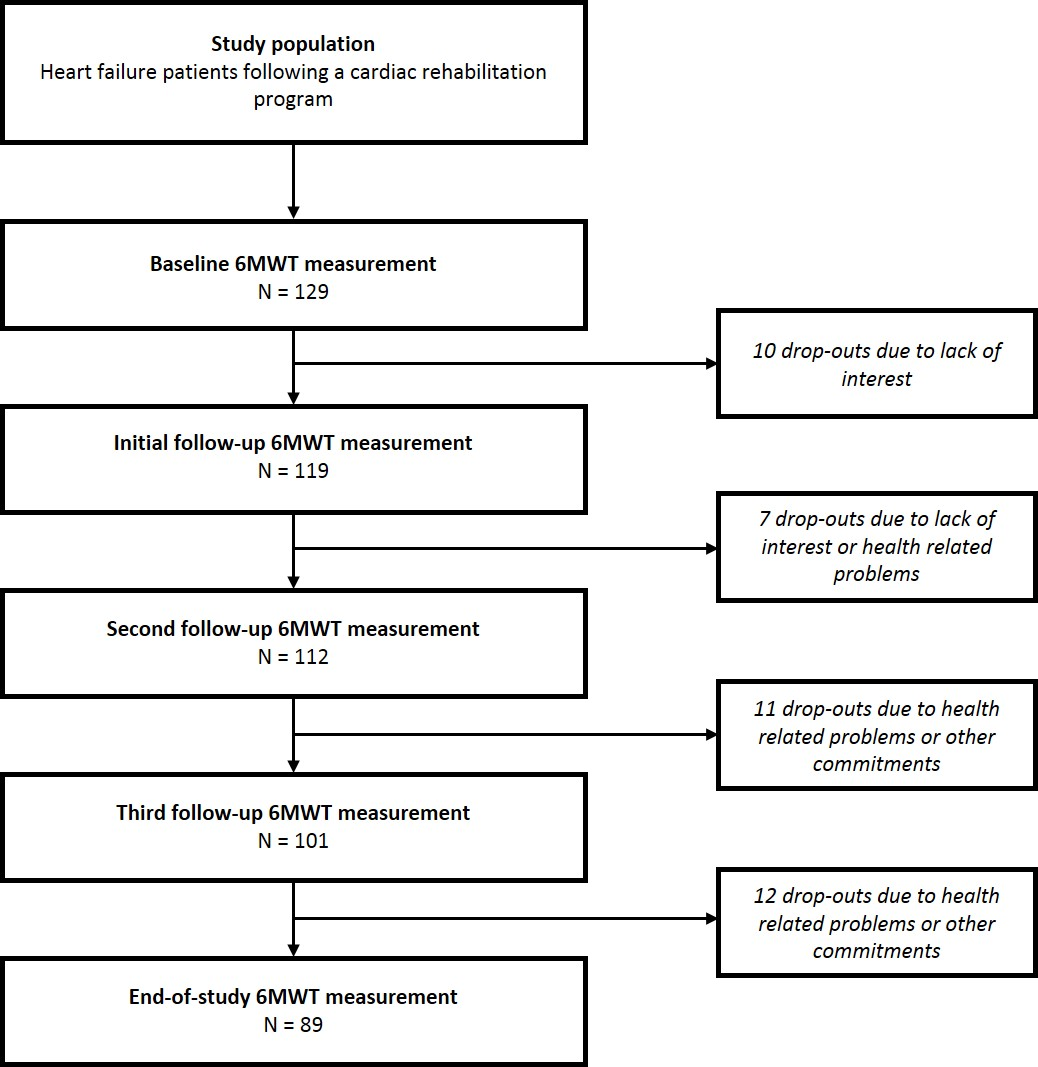

Supplement: Multimedia Appendix 1 [file jmir_v22i5e17326_app1.png]
